# Statins interfere with the attachment of *S. cerevisiae* mtDNA to the inner mitochondrial membrane

**DOI:** 10.1080/14756366.2019.1687461

**Published:** 2019-11-07

**Authors:** Angela Cirigliano, Antonia Amelina, Beatrice Biferali, Alberto Macone, Chiara Mozzetta, Michele Maria Bianchi, Mattia Mori, Bruno Botta, Elah Pick, Rodolfo Negri, Teresa Rinaldi

**Affiliations:** a Department of Biology and Biotechnology “Charles Darwin”, Sapienza University of Rome, Rome, Italy; b Pasteur Institute-Cenci Bolognetti Foundation, Rome, Italy; c Institute of Molecular Biology and Pathology, CNR, Department of Biology and Biotechnology “Charles Darwin”, Sapienza University of Rome, Rome, Italy; d Institute of Molecular Biology and Pathology, CNR, Dipartimento di Scienze Biochimiche “A. Rossi Fanelli”, Sapienza University of Rome, Rome, Italy; e Department of Biotechnology, Chemistry and Pharmacy, University of Siena, Siena, Italy; f Dipartimento di Chimica e Tecnologie del Farmaco, Sapienza University of Rome, Rome, Italy; g Department of Biology and Environment, Faculty of Natural Sciences, University of Haifa at Oranim, Tivon, Israel

**Keywords:** Statins, ergosterol, cholesterol, mitochondrial DNA, myopathy

## Abstract

The 3-hydroxy-3-methylglutaryl-CoA reductase, a key enzyme of the mevalonate pathway for the synthesis of cholesterol in mammals (ergosterol in fungi), is inhibited by statins, a class of cholesterol lowering drugs. Indeed, statins are in a wide medical use, yet statins treatment could induce side effects as hepatotoxicity and myopathy in patients. We used *Saccharomyces cerevisiae* as a model to investigate the effects of statins on mitochondria. We demonstrate that statins are active in *S.cerevisiae* by lowering the ergosterol content in cells and interfering with the attachment of mitochondrial DNA to the inner mitochondrial membrane. Experiments on murine myoblasts confirmed these results in mammals. We propose that the instability of mitochondrial DNA is an early indirect target of statins.

## 1. Introduction

Cholesterol is a vital component of cell membranes, essential for the synthesis of steroid hormones, bile acids, and vitamin D, but high cholesterol levels are associated with an elevated risk of cardiovascular diseases. Cholesterol (ergosterol in yeast) is the end-product of the mevalonate pathway^1^. The enzyme 3-hydroxy-3-methylglutaryl coenzyme A (HMG-CoA) reductase is the rate-limiting step in the synthesis of cholesterol and it catalyses the conversion of HMG-CoA to mevalonate; the mevalonate pathway and the HMG-CoA enzyme, as well as its regulation are conserved from yeast to humans^2^^,^^3^. HMG-CoA is the target of statins^4^, the most prescribed drug class for lowering elevated LDL-cholesterol, generally effective and well tolerated; however, patients can experience muscle adverse effects such as myopathy^5^^,^^6^. Genetic factors^7^ and drug-drug interactions^8^ also contribute as risk factors for the development of muscle side effects in patients^9^^,^^10^.

Mitochondrial dysfunction has been described as responsible for several statins side effects and serious adverse reactions; in particular, various mitochondrial phenotypes were described such as the decrease of respiratory chain function, alteration of calcium homeostasis, ROS production, alteration of mitochondrial volume, however, the primary effect of statins remains to be elucidated^11^^,^^12^. The activity of statins was investigated previously in the yeast *Candida glabrata*^13^ and in *Saccharomyces cerevisiae*^14^; the yeast *S. cerevisiae* is an excellent unicellular model to gain insights into human diseases^15^ in particular, those resulting from impaired mitochondrial function^16–18^, because *S. cerevisiae* is able, utilising fermentable carbon sources to survive without mitochondrial respiration. We previously demonstrated that ketoconazole, which blocks the ergosterol biosynthetic pathway downstream the HMG-CoA reductase, reduced the ergosterol content in yeast cells and induced the loss of mitochondrial DNA; this phenomenon is coupled with a re-localisation of Erg27 enzyme from Lipid Droplets (LDs) to Endoplasmic Reticulum (ER)^19^. Here, we expand the analysis of mitochondrial function and the stability of mitochondrial DNA using statins in *S. cerevisiae*: we showed that these molecules cause a reduction of ergosterol, inducing loss of mitochondrial DNA and resulting in a reduction of mitochondrial respiration/function coupled with the Erg27 re-localisation in ER. In murine myoblasts, upon statins treatment, we also observed a reduction of mitochondrial DNA copy number associated with an impaired differentiation capacity.

## 2. Materials and methods

### 2.1. Yeast strains and growth conditions

The strains used in this study: the *S. cerevisiae* W303 (MAT a, his3–11, ade2–1, leu2–3, −112, ura3–1, trp1–1, can1–100), W303-*ERG27-GFP,* MValC25T (MATa, ade2–1, ura3–52, leu2, kar1–1, syn); the *K. lactis* PM6-7A (MATa uraA1-1 adeT-600^a^), PM6-7A/*Δpda1* (Klpda1::Tn5BLE). The plasmid pVT100U-mitoGFP was used to visualise mitochondria.

Yeast culture media: YPD (1% bacto peptone, 1% yeast extract and 2% glucose), was used as rich medium. YPG (1% bacto peptone, 1% yeast extract and 3% glycerol), was the medium used to verify the respiratory competence of yeast colonies. All media were supplemented with 2.3% bacto agar (Difco) for solid media.

YPD medium supplemented with 0.5 mg/ml of ergosterol. Ergosterol and Tween 80 were dissolved in pure ethanol to final concentration of 10 mg/ml for ergosterol and 42% for Tween80, and steamed at 100 °C for 10 min, always protected from light, before being added to the medium. Yeast cultures were grown and analysed in exponential phase at 28 °C, unless otherwise specified.

### 2.2. C2C12 cell culture

C2C12 myoblasts were grown at 5% CO2 and 37 °C in Dulbecco’s modified Eagle’s medium (DMEM; Gibco) supplemented with 1% penicillin/streptomycin (Gibco) and 10% foetal bovine serum (Gibco). To induce differentiation, cells were cultured with Dulbecco’s modified Eagle’s medium and 2% horse serum (Gibco) for 48 h, when more than 90% of the cells had fused into myotubes. C2C12 were treated for 48 with 25uM Atorvastatin in growth medium and then induced to differentiate for further 48 h either in the presence or absence (ctrl) of the compound.

### 2.3. Rho° production

Strains (W303) devoid of mitochondrial DNA were produced as follows: cells were grown at the density of 1 × 10^6^cells/ml on YPD medium, phosphate buffer pH 6.5 was added in1 ml of culture, at the final concentration of 0.05 M, and ethidium bromide at final concentration of 50 μg/ml. The cultures were incubated at 28 °C for 24 h. Cells were washed twice, plated on YPD and incubated at 28 °C. The absence of mitochondrial DNA was assessed by DAPI staining and absence of growth on glycerol containing medium as a carbon source.

### 2.4. *S. cerevisiae* cell culture

The cells were pre-activated in YPD and YPD rich medium supplemented with different statin up to the stationary phase. Subsequently, the cultures were all diluted to a concentration of 10^4^ cells/ml with the respective media and incubated at 28 °C. The growth trend was monitored with the measurement of the optical density OD600 and/or with the cell count with the Bürker chamber.

### 2.5. Petites assay

Cells were grown in YPD until the stationary phase. At the concentration of 1 × 10^7^cells/ml and 1 × 10^8^cells/ml, 100 cells were spotted in YPD plates and incubated at 28 °C. After two days, colonies were replicated on YPG plates. The lack of rho° cell growth was assessed on glycerol as the carbon source and the absence of mtDNA by 4′,6-diamidino-2-phenylindole (DAPI; Sigma) staining.

### 2.6. Microscopy

Cells were observed with a Zeiss Axio Imager Z1 Fluorescence Microscope with AxioVision 4.8 Digital Image Processing System, the objective lens used was 63× Oil. Filter sets: 38HE (GFP), 43HE (DsRed). Filters for GFP (470/40 nm excitation and 525/50 nm emission) and DAPI (365-nm excitation and 445/450-nm emission), were used Metamorph software (Universal Imaging, West Chester, PA) was used to deconvolute Z-series and treat the images^20^.

### 2.7. Cells treatment

Pravastatin and simvastatin were hydrolysed in ethanolic NaOH [15% (v/v) ethanol and 0.25% (w/v) NaOH] at 60 °C for 1 h. Atorvastatin and rosuvastatin were dissolved in H_2_Odd supplemented with 1% DMSO and 0.9% NaCl. Q10 was hydrolysed in 100% acetone.

Cells were grown to a density of 1 × 10^7^cells/ml in YPD rich medium supplemented with: pravastatin 150 µg/ml, atorvastatin 100 µg/ml, rosuvastatin 50 µg/ml and Q10 10, 50 µg/ml. The cells treated were used for several assays.

### 2.8. Mass spectrometric analyses

Dry cell pellets were deep frozen by immersion in liquid nitrogen and quickly ground to a powder using an IKA A11 basic laboratory mill (IKA, Staufen, Germany). 300 mg of cell pellet were transferred to glass vials with 10 ml Folch solvent (chloroform/methanol 2:1, v/v) containing 0.01% butylated hydroxytoluene followed by the addition of10μl of internal standard (19-hydroxy-cholesterol, 2 mg/ml in methanol) as internal standards (IS). The homogenisation and extraction (60 min) were carried out at room temperature (RT). The extracts were evaporated under a stream of N2then the dry residue was dissolved in1 ml hydrolysis solution containing 5% KOH in (MeOH/H2O, 95:5) and subjected to saponification (1 h, 80 °C). The hydrolysed solution was extracted with 0.5 ml distilled H2O and 4 ml diethyl ether. The ether phase was dried under a stream of N2at RT. The samples were then reconstituted with 25 μl ethylacetate and vortex mixed. The fractions to be analysed were converted to their trimethyl-silylated derivatives by heating with 40 μl BSTFA containing 1% TMCS at 70 °C for 45 min. The sample was dried under a stream of N2. The residue was dissolved in200 ml hexane and the clear hexane phase was transferred into a glass vial for GC–MS injection. GC–MS analyses were performed with an Agilent 6850 A gas chromatograph coupled to a 5973 N quadrupole mass selective detector (Agilent Technologies, Palo Alto, CA, USA).

Chromatographic separations were carried out with an Agilent HP5msfused-silica capillary column (30 m × 0.25 mm i.d.) coated with 5%-phenyl-95%-dimethylpolysiloxane (film thickness 0.25 μm) as stationary phase. Injection mode: splitless at a temperature of 280 °C. Column temperature programme: 120 °C (1 min) then to 320 °C at a rate of 5 °C/min and held for 5 min. The carrier gas was helium at a constant flow of 1.0 ml/min. The spectra were obtained in the electron impact mode at 70 eV ionisation energy; ion source 280 °C; ion source vacuum10-5 Torr. MS analysis was performed simultaneously in TIC (mass range scan from m/z50 to 800 at a rate of 0.42 scans s-1) and SIM mode. For GC-SIM-MS analysis, ion m/z 363 was selected for ergosterol and ion m/z 353 was selected for the internal standard.

### 2.9. O_2_ consumption measurement

Respiration studies were performed using a Clark oxygen electrode (Hansatech Instruments) as described in De Luca et al. (2009)^21^. Cells (1 × 10^8^) were collected, washed with 1 ml sterile water, suspended in 1 ml sodium phosphate buffer (10 mM pH 7.4 containing 4 g l^−1^ glucose) and loaded in the reaction vessel of the previously calibrated oxygen electrod^22^.

### 2.10. Immunofluorescence

For the immunofluorescence on C2C12 cells were fixed with 4% paraformaldehyde in PBS for 20 min at RT, and permeabilized with 0.5% Triton X-100 in PBS. After 1 h incubation with blocking solution (4% BSA in PBS), primary antibody for MyHC (DSHB, clone MF20; 1:50 in blocking solution) was incubated overnight (O/N) at 4 °C. A goat anti-mouse secondary antibody coupled to Alexa Fluor 488 (Thermo Fisher; A21202; 1:250 in blocking solution) was then incubate for 1 h. DNA was counterstained with DAPI (Sigma; D9542). Coverslips were mounted in ProLongTM Diamond Antifade Mountant (Invitrogen; p36970). ImageJ was used to quantify the percentage of nuclei inside the myotubes compared to control (Differentiation index). Images were taken with Nikon eclipse TE300 microscope. Pictures showed in the figures are representative of all examined fields.

### 2.11. Quantitative analysis of mitochondrial DNA

Total DNA was isolated using phenol/chloroform-extraction. mtDNA was quantified by q-PCR using PowerUpSYBR Green PCR Master mix (Applied Biosystems; 4367659) and analysed on a StepOne Plus Real-Time PCR System (Applied Biosystems). To evaluate the amount of mtDNA present per nuclear genome, we quantified the relative copy number differences through the ΔΔCt method^23^, comparing mtDNA and nuclear DNA using the following primers sequences: mtDNA CYTOCHROME C (mt-CO1) FW: 5′-TCATCCCTTGACATCGTGCT-3’ RV: 5′-GTCTGAGTAGCGTCGTGGTA-3’; nuclear DNA ACTIN BETA (ACTB) FW: 5′-CCCTGAGTGTTTCTTGTGGC-3′ RV: 5′-GTCTCCGGAGTCCATCACAA-3′.

## 3. Results

### 3.1. Statins induced a petite phenotype in *S. cerevisiae*

As a potent HMG-CoA inhibitor, statins reduce cellular ergosterol levels also in *S. cerevisiae*. Indeed, the effect of lovastatin on yeast was previously investigated in the *S. cerevisiae* wild type 2180–1 A^14^^,^^24^ and BY4741 (derivative of S288C) model laboratory strains^25^. Similarly, the effect of the additional statins, including rosuvastatin, atorvastatin, simvastatin and fluvastatin was tested in the BY4742 genotype, expressing the human orthologue of HMG-CoA^26^.

Herein, we used *S. cerevisiae* W303 yeast cells to investigate the effects of human HMG-CoA reductase inhibitors; W303 is an excellent model to study sterols because the level of sterols is higher than in S288C and derivative strains^27^^,^^28^.

**Figure 1.**
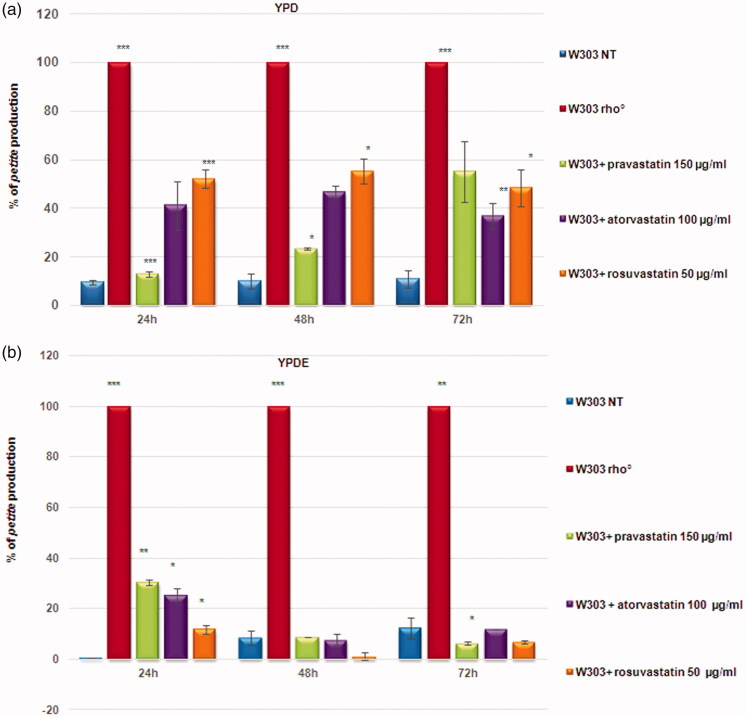
Statins induced a high percentage of *petites* production in yeast cells. (a) Percentage of *petites* production of wild type W303 and W303 rho° grown in YPD medium and wild type W303 grown in YPD medium with pravastatin 150 µg/ml, atorvastatin 100 µg/ml and rosuvastatin 50 µg/ml. (b) The *petite* phenotype is suppressed by ergosterol. The same statins treatments as in (a) didn’t show a *petite* phenotype production in cells grown in a medium supplemented with ergosterol (0,5 mg/ml, YPDE). Cultures grown 24, 48 and 72 hours were plated on YPD plates and petites were counted after a replica plating on glycerol containing medium. Data are the average of 3 independent experiments and standard deviation is indicated. The asterisks indicate significant modulations (*p* < 0.05) according to Student’s T-test.

To assess the effect of statins on mitochondria, we selected pravastatin, rosuvastatin, and atorvastatin for our studies, at non-toxic concentrations (Supplementary Figure 1) previously used to study the effect of statins on yeast^26^. The treatment of wild type W303 cells with statins on agar YPD, led to the production of high percentage of small colonies. These colonies are referred to as *petites*, a phenotype which indicates a mitochondrial dysfunction, hence we assessed the effects of statins on yeast mitochondrial function using a *petite* assay (Supplementary Figure 2). The *petite* phenotype refers to the size of a *S. cerevisiae* colony with rearrangements (rho^−^) or the lack of mitochondrial DNA (rho°). We monitored *petite* production plating the cultures at 24, 48, 72 h of growth. At the concentration used, the statins induced a high rate of *petite* phenotype, especially atorvastatin and rosuvastatin (Figure 1(A)), demonstrating that the statins induced a defect in mitochondrial function, similar to ketoconazole.

We previously show that in yeast, the presence of ergosterol (0.5 mg/ml) in growth medium suppressed the *petite* phenotype induced by ketoconazole^19^. We verified if the generation of *petite* colonies in presence of statins could be counteracted by adding ergosterol, at a concentration used to suppress the ketoconazole effects. Indeed, the percentage of *petite* was very low in presence of ergosterol, in similar to the wild type strain with functional mitochondria (Figure 1(B)).

Because treated cells showed different size, we verified cellular vitality by counting cells number to construct growth curves, which is not consider with optical density measure (Supplementary Figure 3(A)). Of note, treatments of W303 with statins resulted in intermediate growth curves, located between the non-treated W303 strain and the strain devoid of mitochondrial DNA (W303 rho°), while the presence of ergosterol in the medium restores a wild type growth in statin treated cells and in W303 rho° (Supplementary Figure 3(B)). These results suggest that statins treatment induced mitochondrial dysfunction in *S. cerevisiae* by lowering ergosterol content. To verify this hypothesis, we measured the content of ergosterol in W303 wild type cells treated with statins, using, as a control, W303 rho° strain, which has a low ergosterol content^17^. Ergosterol content was measured in yeast cells treated with statins by gas chromatography-mass spectrometry (GC-MS) (Figure S4). The results revealed that ergosterol content was significantly lower in statin-treated cells, compared with the non-treated wild type strain. These results are in agreement with a previous study in *Candida glabrata,* demonstrates that simvastatin reduces ergosterol quantities in cells^17^.

### 3.2. The petite colonies induced by statin treatment contain rho° cells, devoid of mitochondrial DNA

**Figure 2.**
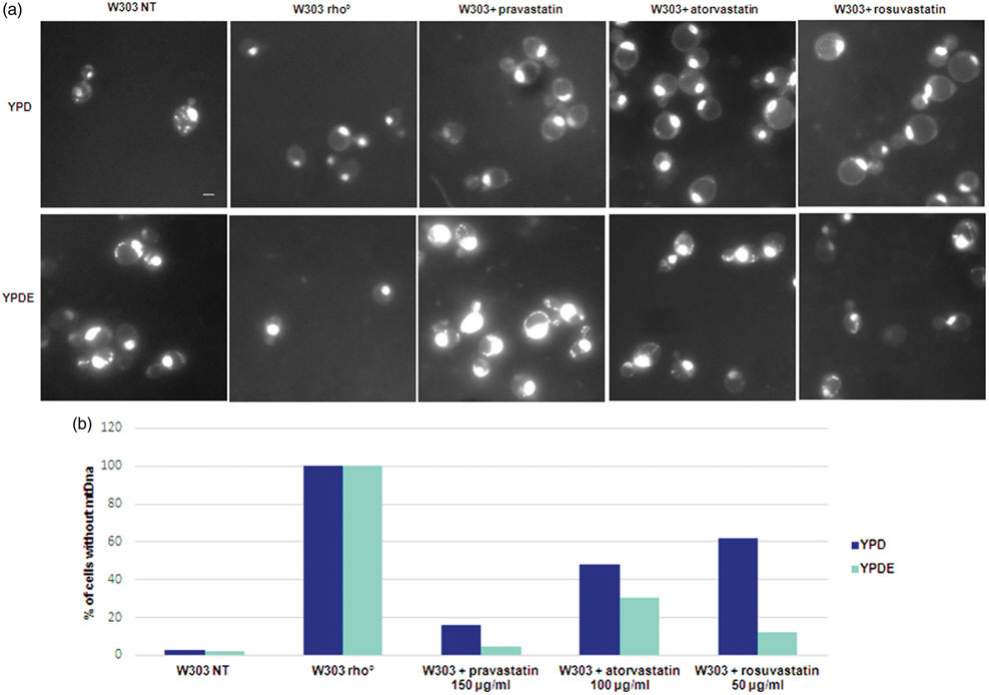
Statins treatments induced the loss of mitochondrial DNA and the ergosterol suppressed this phenotype. (a) DAPI staining of cells presented in Fig. 1; wild type W303, W303 rho° and wild type W303 treated with pravastatin 150 µg/ml, atorvastatin 100 µg/ml and rosuvastatin 50 µg/ml grown in YPD and YPDE media (YPD supplemented with 0,5mg/ml ergosterol). Bar: 2 µm. The magnification is the same in each picture. (b) The histogram shows the percentage of cells without mitochondrial DNA. An average of 300 cells were counted.

To establish if statins act directly on the stability of mitochondrial DNA, because of low ergosterol content, we visualised mitochondrial DNA from *petite* colonies by DAPI staining. Results, reported in Figure 2(A), showed that wild type cells contained mitochondrial DNA, W303 rho° cells had only nuclear DNA and wild type cells treated with statins showed a high percentage of cells devoid of mitochondrial DNA. The quantitative analysis of rho° cells generated by statins is reported in Figure 2(B): histograms report the percentage of cells without mitochondrial DNA. The same experiment was performed in parallel with cultures grown in YPDE medium.

**Figure 3.**
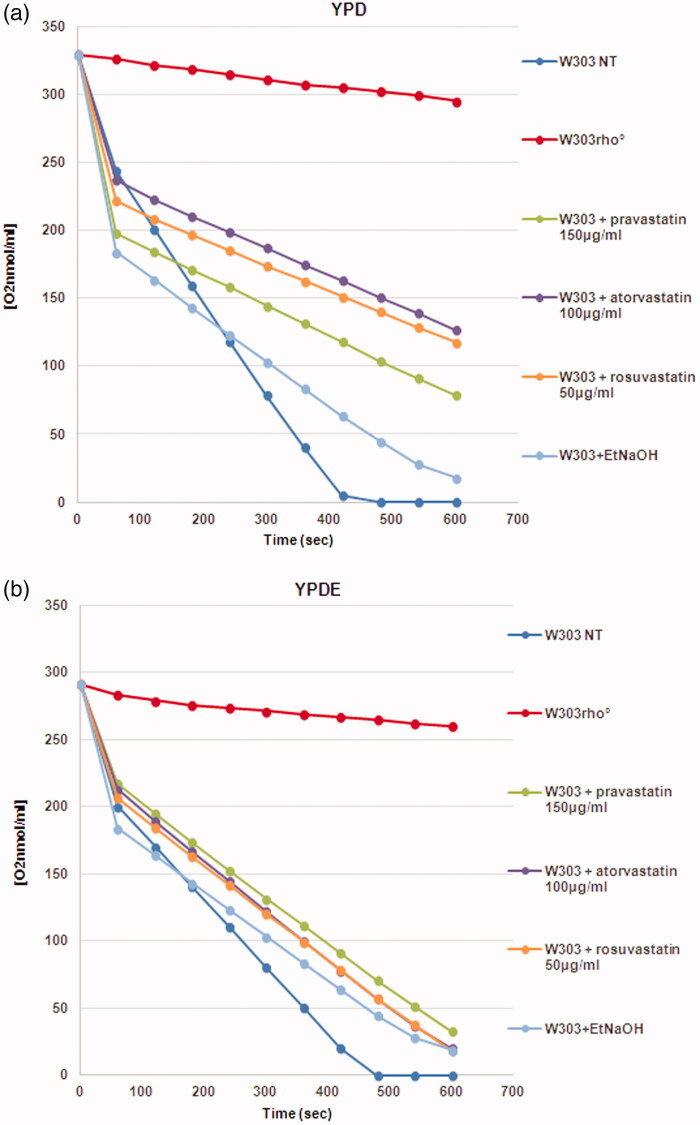
The oxygen consumption rate of yeast cells treated with statins is low compared to the wild type strain. (a) Oxygen consumption of W303, W303 rho° and W303 cells treated with statins in YPD medium showed low oxygen consumption in cells treated with statins; (b) The respiration rate is higher when the same cultures were grown in YPD medium supplemented with ergosterol. The oxygen consumption rate is expressed as O_2_ nmol/ml, and the respiration rates was normalized for cell number.

Results demonstrated that statins induced the loss of mitochondrial DNA and that the presence of ergosterol suppressed this phenotype; we also verified that the colonies with mitochondrial DNA were able to grow on glycerol medium (not shown). This result indicate that loss of mitochondrial DNA was correlated to low concentration of ergosterol in the mitochondrial membranes and that the exogenous addition of ergosterol was sufficient to stabilise the mitochondrial DNA, counteracting the statins effect. We then measured the oxygen consumption of cells treated with statins; Figure 3(A) reports that the respiration rate of wild type strain treated with statins is lower if compared with the non-treated wild type cells; when the same cultures were grown in YPD medium supplemented with ergosterol, the respiration rate was higher, indicating that the presence of ergosterol compensate for the effect of statins (Figure 3(B)). This result suggests an impairment of the respiratory chain function when cells were treated with statins (see Discussion).

### 3.3. Statins treatment resulted in mitochondrial morphology defects

**Figure 4.**
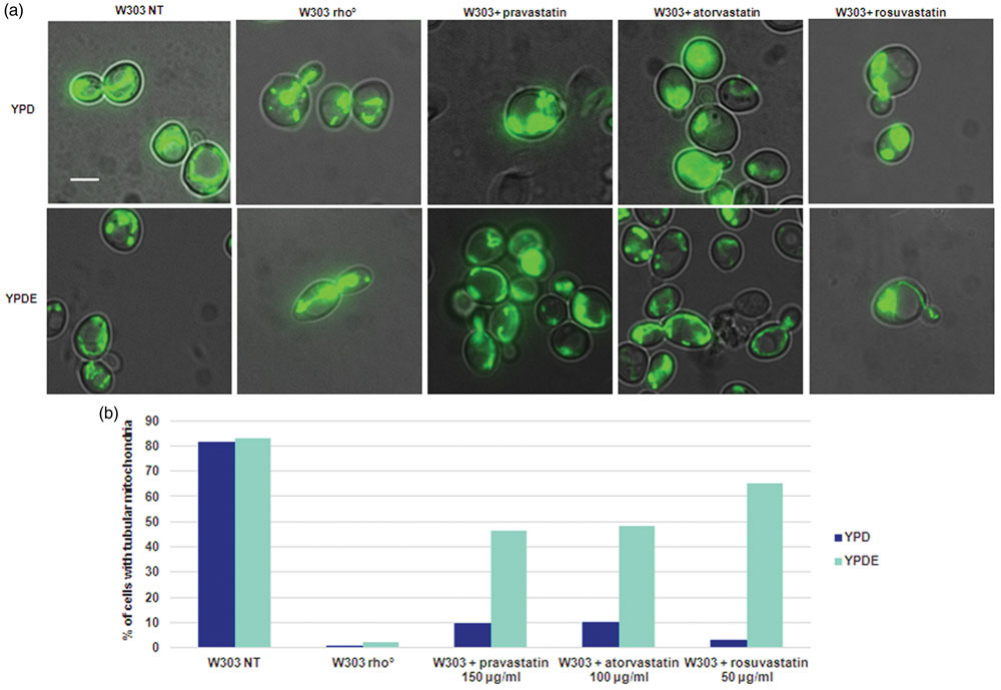
Statins treatments in S. cerevisiae results in an aberrant mitochondrial morphology phenotype, similar to that of rho° cells and this phenotype is suppressed by ergosterol. (a) W303 was treated with pravastatin 150 µg/ml, atorvastatin 100µg/ml and rosuvastatin 50 µg/ml grown in YPD and YPDE media in exponential growth phase; mitochondria were visualized with mitoGFP. (b) The histogram shows the percentage of cells with tubular mitochondria. As a control, W303 rho° strain was used. Bar: 2 µm. The magnification is the same in each picture.

In yeast, cells lacking mitochondrial DNA or a conditional mutant (ERG27) of the ergosterol pathway show defects in mitochondrial morphology^19^ and a down regulation of the ergosterol genes results in mitochondrial membrane alteration^29^^,^^30^. We therefore analysed mitochondrial morphology after statins treatment (Figure 4(A)). Accordingly, wild type strain transformed with a vector expressing the mitoGFP for green fluorescent labelling of mitochondria in living cells, and mitochondrial morphology was assessed upon treatment with statins at exponential phase, in glucose-containing medium with or without ergosterol. By fluorescence microscopy, we observed a relevant percentage of aggregated vs tubular mitochondria in wild type strain treated with statins. This phenotype is reminiscent of mutants lacking mitochondrial DNA, or of W303 rho° strain^19^. The addition of ergosterol partially suppressed the aggregated mitochondrial morphology phenotype, partially restoring a tubular mitochondrial structure (Figure 4(B)). By staining cells with DAPI, we were able to verify the presence of mitochondrial DNA in the strains expressing mitoGFP, treated with statins, and supplemented with ergosterol (Supplementary Figure 5).

### 3.4. Statins induced a cellular re-localisation of Erg27, a sensor of mitochondrial dysfunction

**Figure 5.**
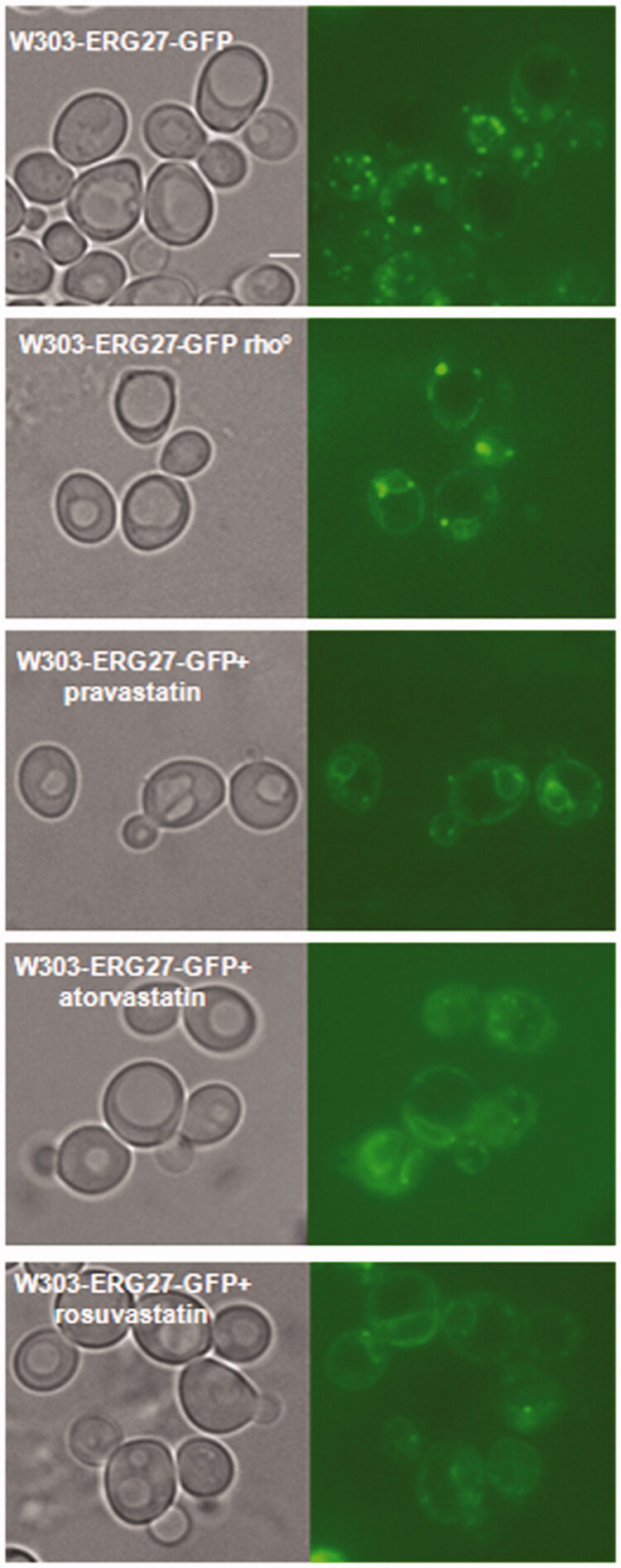
Statins treatments induced the re-localization of Erg27, a sensor of mitochondrial state, from lipid droplets to nuclear and cortical ER. The Erg27 localization in wild type cells is predominantly in lipid droplets, while in rho° cells the enzyme is mainly in nuclear and cortical endoplasmic reticulum. Wild type W303 with the ERG27-GFP fusion integrated in the genome was visualized at fluorescence microscopy after statins treatments, pravastatin 150µg/ml, atorvastatin 100µg/ml and rosuvastatin 50µg/ml in exponential growth phase in YPD medium. Bar: 2 µm. The magnification is the same in each picture.

Erg27 is an essential enzyme of the ergosterol biosynthetic pathway that plays as a sensor of mitochondrial function: in wild type cells the enzyme is mainly found in Lipid Droplets (LDs), while in cells with a block of respiration or in rho° cells, it moves to the nuclear and cortical Endoplasmic Reticulum (ER)^19^. To assess a possible mitochondrial defect, we determined the cellular localisation of Erg27 following statins treatment. Wild type W303 and W303 rho° strains, harbouring the *ERG27-GFP* fusion integrated in the genome, were treated with statins and analysed by fluorescence microscopy. Results reported in Figure 5 showed that in wild type cells, Erg27-GFP decorated lipid droplets, while in cells treated with statins, Erg27-GFP re-localised in nuclear and cortical ER, as in W303 rho° cells. The re-localisation of Erg27-GFP demonstrated that statins, similarly to a block of respiration, induced the movement of Erg27 from lipid droplets to the endoplasmic reticulum.

### 3.5. Effect of statins on the respiratory obligate yeast Kluyveromyces lactis

The yeast *Kluyveromyces lactis* has a metabolism similar to that of human cells and the regulation between fermentative and respiratory metabolism depends on oxygen availability; moreover, in *K. lactis*, as in human cells, cells cannot survive without mitochondrial DNA^31^. We analysed the effect of statins on *K. lactis* growth, in order to enlighten the effects of statins on respiration in a yeast that could mimic the human cell behaviour. The K. *lactis Δpda1*, a mutant strain lacking the pyruvate dehydrogenase E1α subunit^19^, was used to mimic the growth behaviour of a *S. cerevisiae* rho° strain. Statins treatments resulted in a slight delay in growth, intermediate between the untreated wild type and the *Δpda1* mutant strain with a defective respiration (Supplementary Figure 6). This experiment demonstrated that statins are effective also in a yeast unable to lose spontaneously mitochondrial DNA, as for human cells.

### 3.6. Effect of statins in a yeast model of human MELAS mitochondrial disease

Statins are not recommended for patients affected by mitochondrial diseases^32^ and patients with Mitochondrial Encephalomyopathy, Lactic Acidosis and Stroke-like episodes (MELAS) experienced myotoxicity^33^^,^^34^. A yeast model for MELAS mitochondrial disease exists: the strain C25T containing the C25T mutation in the mitochondrial tRNALeu (UUR), is the yeast counterpart of the human mitochondrial C3256T mutation in the tRNALeu (UUR) gene, identified in MELAS patients^35^. The C25T mitochondrial mutation induces a reduced growth on a respiratory carbon source and shows mitochondrial morphology defects^36^. In order to verify if the effect of statins treatment could be additive on the yeast MELAS phenotypes, we followed the growth of C25T cells with statins (Supplementary Figure 7). The treatment with statins causes a mild effect on growth in exponential phase, intermediate between rho° cells and the non-treated mutant. Of note, pravastatin seems to exert a greater effect, inducing a lag phase in growth. This experiment showed that statins exacerbated the mitochondrial dysfunction of a yeast strain with impaired mitochondrial function.

### 3.7. *In vitro* effects of statins on C2C12 murine cells

**Figure 6.**
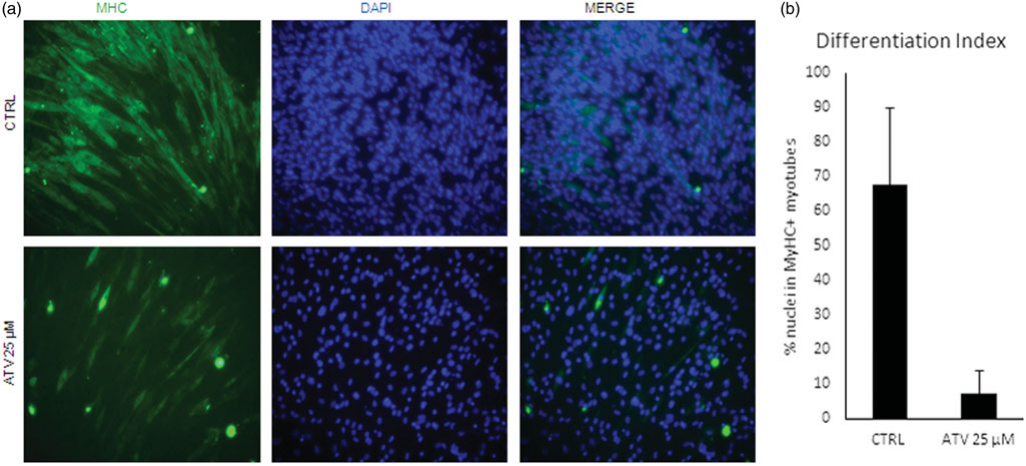
Statins block C2C12 differentiation and induce loss of mtDNA. (a) C2C12 were treated for 48 with 25uM atorvastatin (ATV) in growth medium and then induced to differentiate for further 48h either. Non-treated cells were used as a control (CTRL). Representative images of immunofluorescence for Myosin Heavy Chain (MyHC, green) are shown. Nuclei were counterstained with DAPI (blue). (b) Graph shows the quantification of the percentage of nuclei inside MyHC positive myotubes (Differentiation index). Data are presented as the average of n=3 replicates +/- SD. Significance has been assessed by t-test (*p < 0.05).

In light of the evidence that statins assumption is often associated with muscle-related side effects, such as fatigue, muscle pain and muscle weakness, we sought to verify the effect of statins on a well-established *in vitro* model of myogenesis, the C2C12 murine myoblasts cell line^37^. To this end, we assessed the differentiation capacity of C2C12 upon atorvastatin (25 µM) treatment, as compared to untreated cells (Ctrl) by immunofluorescence for Myosin heavy chain, a marker of terminal myogenic differentiation. As shown in Figure 6, atorvastatin dramatically reduced C2C12 myogenic differentiation (Figure 6(A)), as quantified evaluating differentiation index (Figure 6(B)). To verify if the observed differentiation defects were also accompanied with mitochondrial dysfunction, we measured the mitochondrial DNA content by qRT-PCR (see Methods). The threshold of amplification between mtDNA (mt-CO1 gene) and nuclear DNA (using ACTB as reference) was measured; the average of ratio of treated/non-treated was 0.96 ± 0.015 (mean ± standard deviation) (mean of *n* = 4 replicates), indicated that atorvastatin induced a dose-dependent reduction of mitochondrial DNA, in agreement with data obtained in yeast cells.

## 4. Discussion

Statin intolerance is often a consequence of statin-associated myopathy^38^^,^^39^; hence, it is fundamental to understand the molecular effect of statin in the cells. Yeast is recognised as a good model to study the cellular mechanisms of mitochondrial human diseases and *S. cerevisiae* is unique among yeasts because it is able to survive with non-functional mitochondria^17^. We confirmed here that statins act as cholesterol lowering drugs in *S. cerevisiae* as was previously published^14^; mass spectrometry analysis revealed a lower ergosterol content in statins treated cells than the wild type strain (Supplementary Figure 1); in yeast cells treated with statins, the Erg27 enzyme localised in the endoplasmic reticulum indicating low level of ergosterol, because in cells with normal ergosterol content, the enzyme is localised in lipid droplets^19^ (Figure 5). Of note, statin treatments resulted in W303 growth curves rates intermediate between the non-treated strain (W303) and the strain devoid of mitochondrial DNA (W303 rho°), while the supply of ergosterol in the medium restores a wild type growth in statin treated cells and in W303 rho° (Supplementary Figure 3(B)). Also, the growth curves of the yeast *K. lactis*, which has a metabolism more similar to human cells, revealed that statins slightly impair the growth, as in *S. cerevisiae* (Supplementary Figure 6). Finally, we treated with statins a yeast model of human MELAS disease^18^. Statins are not administered to MELAS patients because they precipitate myopathic symptoms^40^; indeed, we found an effect on C25T strain growth, indicating an additive negative affect of stations if associated with a MELAS mutation (Supplementary Figure 7).

Taking together these results indicate that *S. cerevisiae* is a suitable model to investigate the mitochondrial effects of statins and also to enlighten the regulation of ergosterol pathway based on the dynamic localisation of Erg enzymes in the cell. Another case of relocalization of an enzyme of the ergosterol biosynthetic pathway was previously described: Erg1 shows a dual localisation both in the ER and LDs, but it is redistributed from LDs to ER in low iron condition in the cells^41^. The cross talk between the mitochondrial respiration and the ergosterol biosynthetic pathway is still poorly understood^19^.

Statins induce a *petite* phenotype (Figure 1(A) and Supplementary Figure 2), caused by the loss of mitochondrial DNA (Figure 2A); *S. cerevisiae* is able to survive without mitochondrial DNA (rho° cells) but can also maintain rearrangements of mitochondrial DNA (rho^−^ cells), induced by chemical agents or by genetic mutations^42^. The lack of mitochondrial DNA in statin treated cells, suggested an effect of statins in the stability of mitochondrial DNA and not in mitochondrial DNA transcription/replication/recombination. The percentage of treated cells showing cells devoid of mitochondrial DNA (Figure 2(B)) fits well with the percentage of *petite* colonies (Figure 1(A)).

The supplementation of ergosterol in the growth medium was able to suppress the mitochondrial DNA loss (Figures 1(B) and 2(A)), demonstrating that a correct amount of ergosterol is essential for mitochondrial function.

To date, a demonstration of how mitochondrial DNA molecules are attached to the inner mitochondrial membrane is still lacking, but lipid rafts, membrane microdomains enriched in cholesterol and sphingolipids^43^ are essential to maintain mitochondrial DNA in human cells^44^. Furthermore, the maintenance of mitochondrial nucleoids is coordinated with the mitochondrial fusion and fission machinery to ensure the distribution of mitochondrial DNA in the cells^45^; of note, the mitochondrial protein ATAD3, which binds cholesterol in the inner mitochondrial membrane, has been proposed to have a central role in nucleoid organisation^46–49^.

The statins investigated in this work showed different behaviours: pravastatin produced less *petites*, it is less toxic, but the ergosterol content is comparable to other statins, and the respiration is more similar to the wild type strain. Rosuvastatin produced the higher percentage of *petites* and it is more toxic, and the respiration is similar to rho° cells. Atorvastatin is intermediate between pravastatin and rosuvastatin. The same behaviour is observed when *K. lactis* was treated with the statins.

An extensive literature reports defects in respiration in mammalian cells treated with statins, by measuring the activity of respiratory chain complexes^50^. We hypothesise that the mitochondrial defects described in literature can be reconciled/explained by the low levels of mitochondrial DNA copy number, that imbalance the ratio between mitochondrial and nuclear-encoded subunits of the respiratory complexes, resulting in an altered respiration rate. Indeed, yeast cells treated with statins showed a respiration rate lower than the wild type strain but, when the same cultures were grown in YPD medium were supplemented with ergosterol, the respiration rate was higher (Figure 3). Of note, the complex II is the only to be entirely nuclear encoded (i.e. this complex does not contain mitochondrial encoded subunits); indeed, the complex II resulted unaffected by simvastatin treatment on skeletal muscle^51^^,^^52^; on the contrary, the other respiratory complexes contain nuclear and mitochondrial- encoded subunits are affected by statins^42^; we hypothesise that a reduced level of mitochondrial DNA could results in an imbalance of the nuclear and mitochondrial encoded subunits, causing an altered respiration rate.

Of note, skeletal muscle biopsies from patients diagnosed with myopathies induced by statins, were used to quantify mitochondrial DNA relative to nuclear DNA (mitochondrial DNA content) by qPCR, and it was found that skeletal muscle mitochondrial DNA is decreased in patients with statin-induced myopathy^53^^,^^54^. In agreement with this, we observed a small but significant reduction of mitochondrial DNA in C2C12 myoblasts after statin treatment. This reduction could explain why muscle cells are more sensitive to statins than other cells, resulting in muscle-related side effects. Indeed, mitophagy has been reported to be essential for proper muscle differentiation^55^ and during muscle differentiation, 4 times more DNA and 8 times more respiratory complexes are needed compared to other cells^56^. Our results support the notion that the amount of mitochondrial DNA is critical in muscle differentiation because after degradation of mitochondria, new mitochondrial DNA synthesis and new membranes are needed to maintain functional myofibers. The statins-induced reduction of the amount of cholesterol necessary to maintain mitochondrial DNA, and for membrane construction, might be the reason why chronic statins treatment induces muscle-related side effects. Accordingly, in presence of statins the differentiation from myoblast to myotubes is dramatically reduced (Figure 6).

In conclusion, we have used *S. cerevisiae* as a model to study the mechanism of statin side effects on mitochondria, and we confirm that a relevant effect of low cholesterol level induced by statins is on mitochondrial DNA stability, explaining why muscle cells are more prone to statins side effects.

## Supplementary Material

Supplemental Material
