# Supplementary material for: Statins interfere with the attachment of S. cerevisiae mtDNA to the inner mitochondrial membrane
Source: J Enzyme Inhib Med Chem. 2019 Nov 7;35(1):129–38. doi: 10.1080/14756366.2019.1687461 (PMC6844431; doi:10.1080/14756366.2019.1687461)

Statins interfere with the attachment of *S.cerevisiae* mtDNA to the inner mitochondrial membrane

Angela Cirigliano[a], Antonia Amelina[a], Beatrice Biferali[a,c], Alberto Macone[b,d], Chiara Mozzetta[a,c], Michele Maria Bianchi[a], Mattia Mori[e], Bruno Botta[f], Elah Pick[g], Rodolfo Negri[a,c] and Teresa Rinaldi[a,b,] *

[a] Department of Biology and Biotechnology "Charles Darwin", Sapienza University of Rome, Piazzale A. Moro 5, 00185 Rome, Italy.

[b]Pasteur Institute-Cenci Bolognetti Foundation, Viale Regina Elena, 291, 00100 Rome, Italy.

[c] Institute of Molecular Biology and Pathology, CNR, Department of Biology and Biotechnology "Charles Darwin", Sapienza University of Rome, Piazzale A. Moro 5, 00185 Rome, Italy.

[d] Institute of Molecular Biology and Pathology, CNR, Dipartimento di Scienze Biochimiche "A. Rossi Fanelli", Sapienza University of Rome, Piazzale A. Moro 5, 00185 Rome, Italy.

[e] Department of Biotechnology, Chemistry and Pharmacy, University of Siena, via Aldo Moro 2, 53100 Siena, Italy.

[f] Dipartimento di Chimica e Tecnologie del Farmaco, Sapienza University of Rome, Rome, Piazzale A. Moro 5, 00185 Rome, Italy.

[g] Department of Biology and Environment, Faculty of Natural Sciences, University of Haifa at Oranim, Tivon, Israel.

* Corresponding author at: Piazzale A. Moro 5, 00185 Rome, Italy.

E-mail address: teresa.rinaldi@uniroma1.it (T. Rinaldi)

Supplementary data

Supplemental Figure 1. Dose-dependent growth profiles obtained by treating yeast cells with statins. The same number of W303 wild-type cells were supplemented with pravastatin (A), simvastatin (B), atorvastatin (C), and rosuvastatin (D) at different concentrations. In E the statin concentrations chosen for this study.

NT indicates non-treated. The simvastatin was excluded in this study because of its toxicity.

A

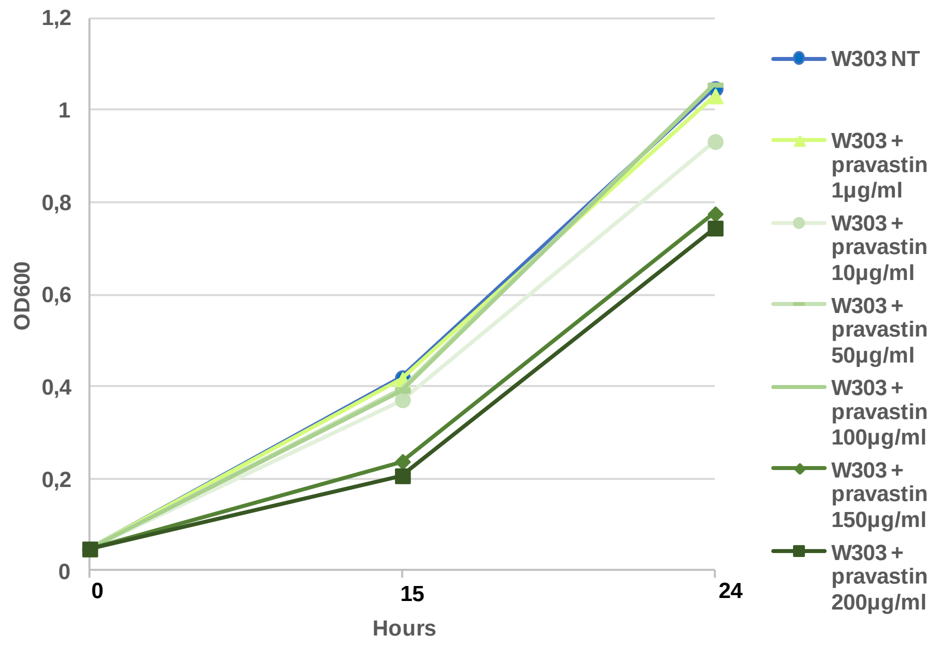

B

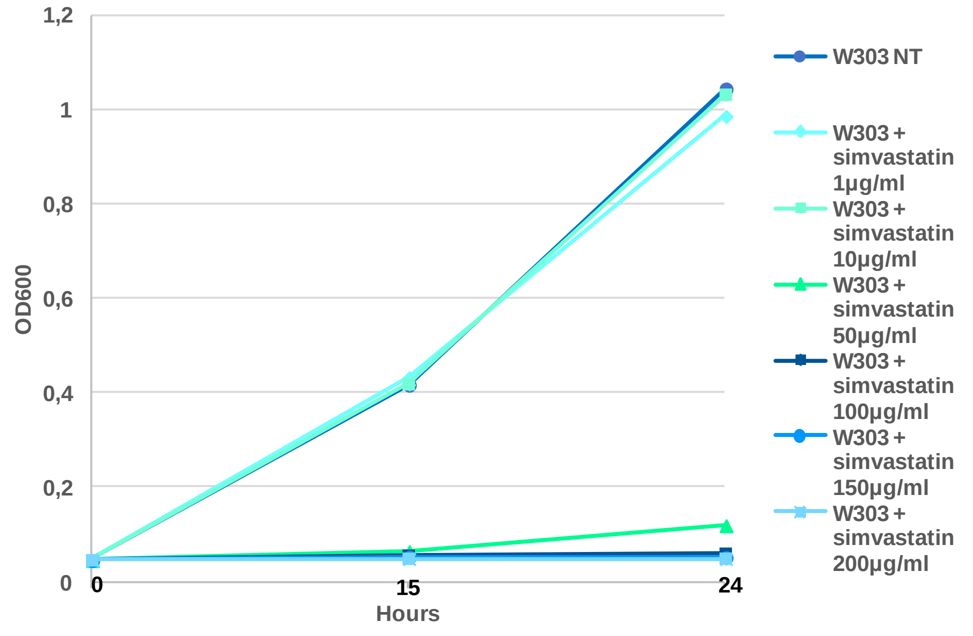

C

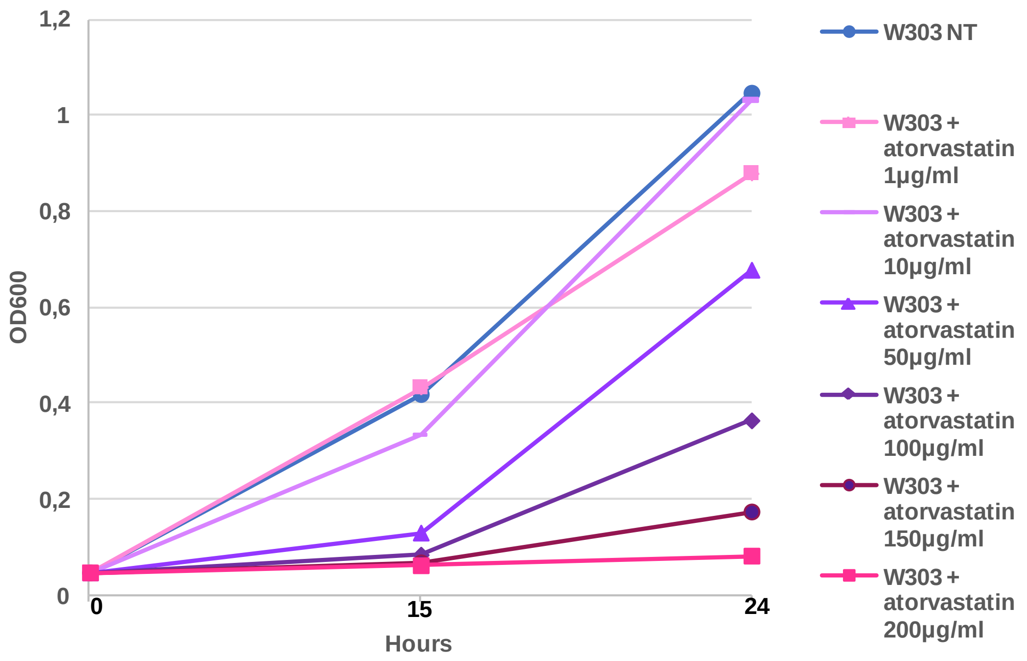

1,2
1
0,8
0,6
0,4
0,2
0
OD600
0
15
24
Hours
W303 NT
W303 + atorvastatin 1µg/ml
W303 + atorvastatin 10µg/ml
W303 + atorvastatin 50µg/ml
W303 + atorvastatin 100µg/ml
W303 + atorvastatin 150µg/ml
W303 + atorvastatin 200µg/ml

D

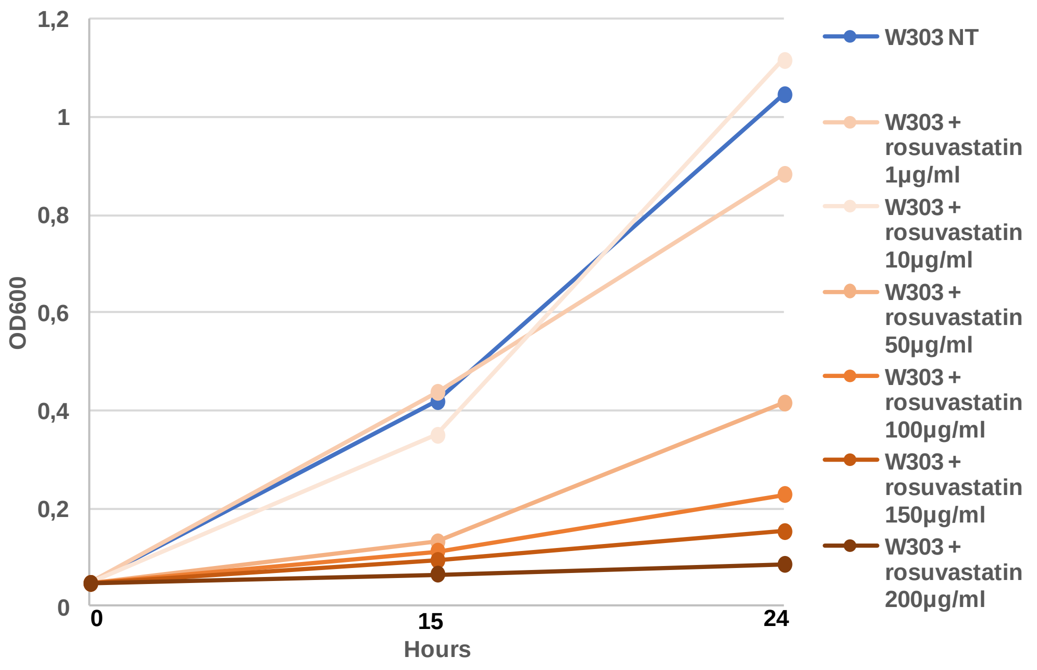

1,2
1
0,8
0,6
0,4
0,2
0
OD600
0
15
24
Hours
W303 NT
W303 + rosuvastatin 1µg/ml
W303 + rosuvastatin 10µg/ml
W303 + rosuvastatin 50µg/ml
W303 + rosuvastatin 100µg/ml
W303 + rosuvastatin 150µg/ml
W303 + rosuvastatin 200µg/ml

E

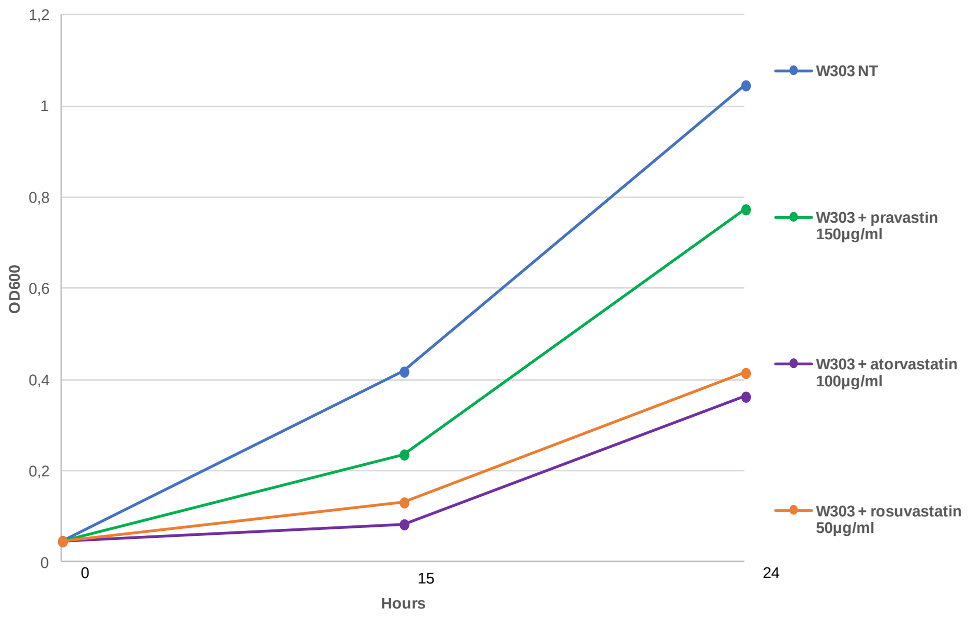
1,2
1
0,8
0,6
0,4
0,2
0
OD600
0
15
24
Hours
W303 NT
W303 + pravastin 150µg/ml
W303 + atorvastatin 100µg/ml
W303 + rosuvastatin 50µg/ml

Supplemental Figure 2. The *petite* phenotype refers to the size of a *S. cerevisiae* colony with rearrangements (rho$^-$) or the lack of mitochondrial DNA (rho°).

Consequently, the absence of respiration results in a small colony compared with normal size colony with functional mitochondria. Moreover, when the wild type yeast has a ade2$^-$ genotype, wild type colonies are red because of the accumulation of the Ade2 substrate, one enzyme of the adenine biosynthetic pathway. This pathway has an Ade2 upstream mitochondrial step, which is nonfunctional in *petites*, resulting in colonies with a white colour. The visualization of red/white colonies facilitates the screening of *petites* production. YPD, complete medium with glucose as carbon source. YPG, complete medium with glycerol as only respirable carbon source. W303 wild type cells, with or without statin, were plated on YPD (left) and after three days replica plated in YPG plates (right). The *petites* colonies (white) were not able to grow with glycerol as carbon source as indicated by white arrows, as an example. To know if the mitochondrial DNA is rearranged (rho$^-$) or absent (rho°), DAPI staining was performed.

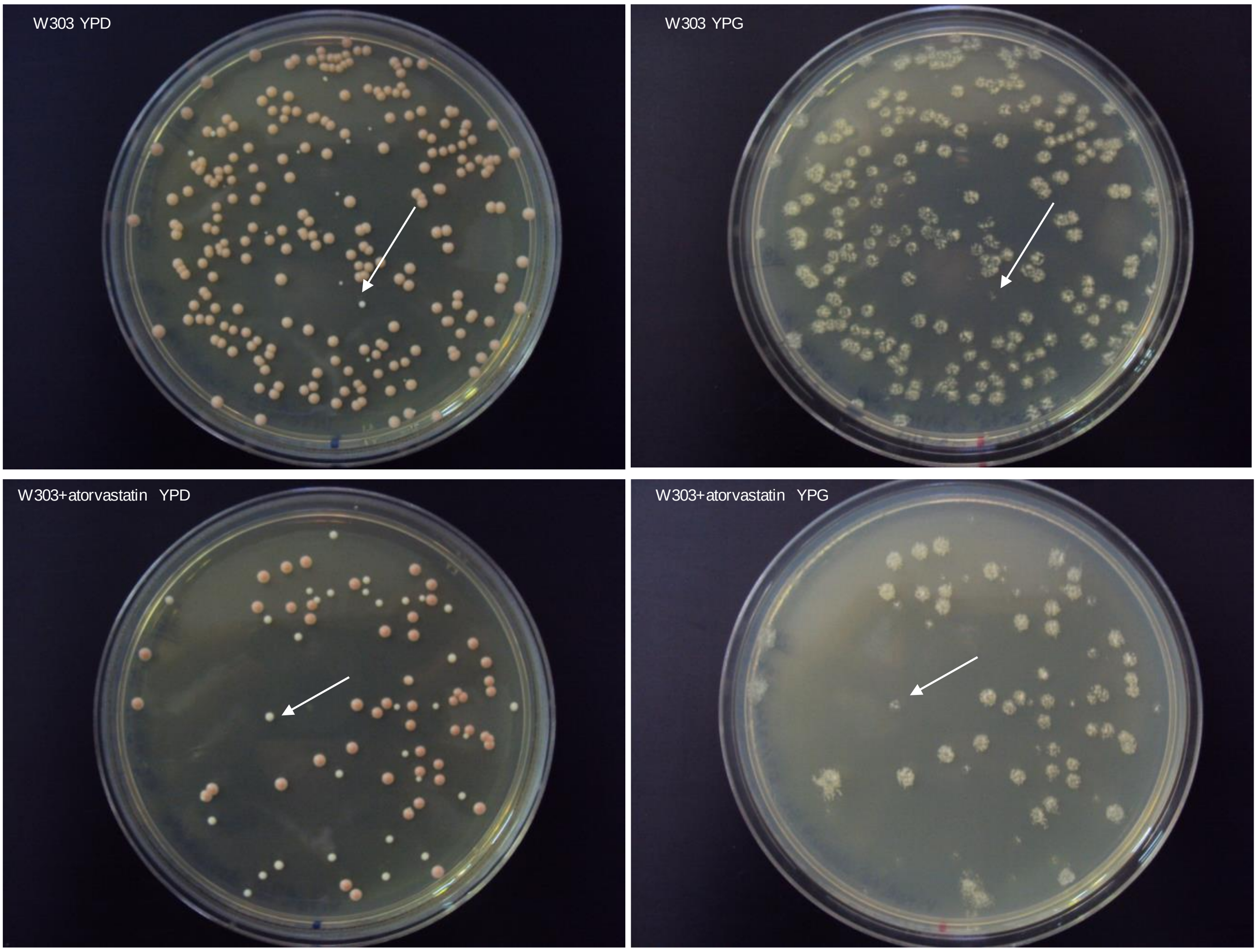

Supplemental Figure 3. Growth curves of cells. (A) Statins treatments slightly affect the growth of cells at the concentrations used. Of note, the statins treatments result in W303 growth curves intermediate between the non-treated strain (W303) and the strain devoid of mitochondrial DNA (W303 rho°). (B) The presence of ergosterol in the medium restores a wild type growth in statin treated cells and in W303 rho°. Yeast growth curves representing the number of cells/ml of cultures at four different time points

A

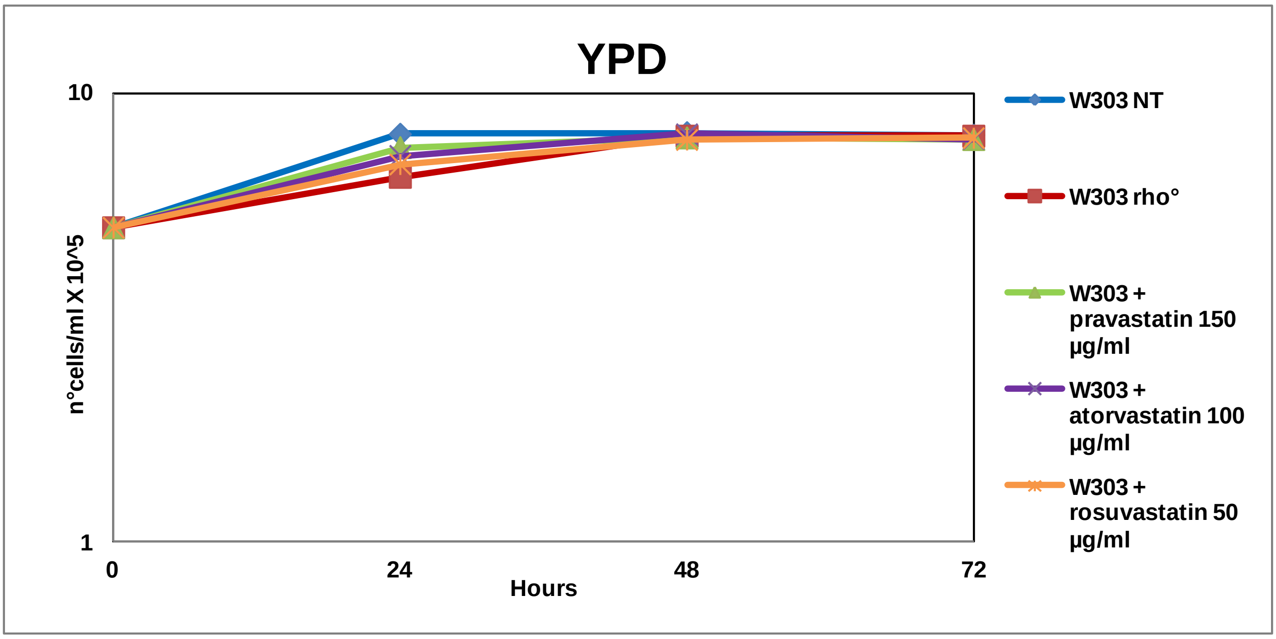

B

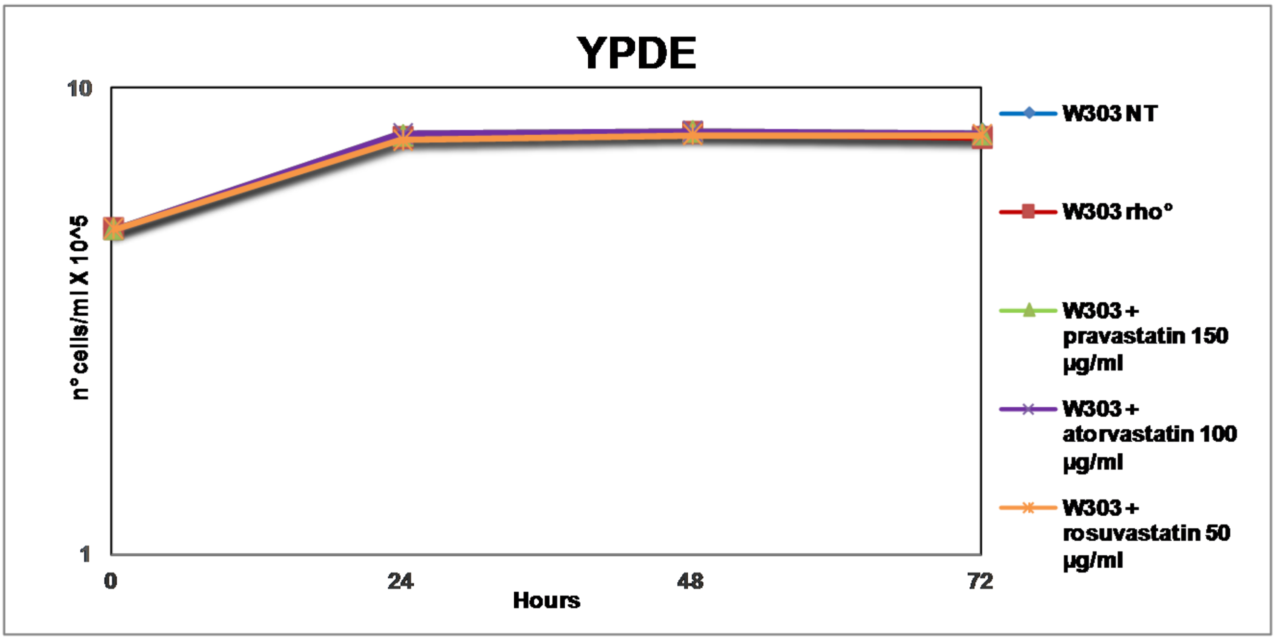

Supplemental Figure 4. Ergosterol is less abundant in yeast cells treated with statins, compared to the not treated wild-type strain. Gas chromatography-mass spectrometry analysis was performed to measure the ergosterol area/internal standard area. Data are the average of 3 independent experiments and standard deviations are indicated. The asterisks indicate significant modulation (*** p<0.005) according to Student's T-tests.

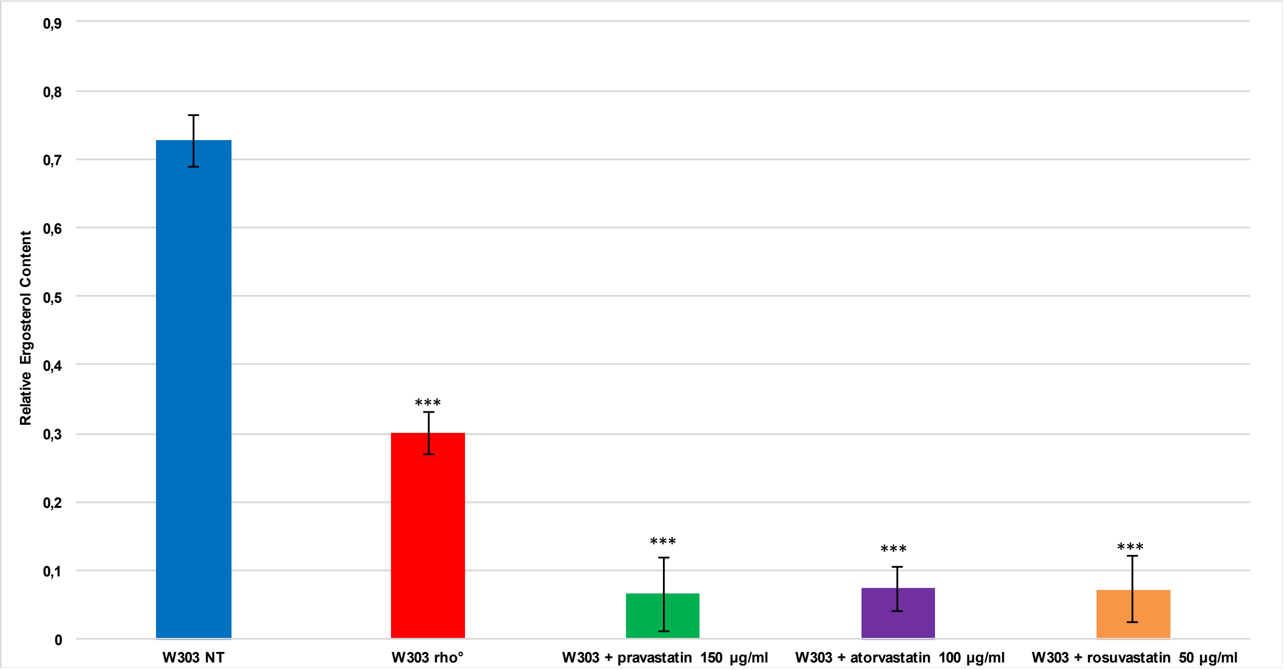

Supplemental Figure 5. The presence of ergosterol in the medium allows wild type cells treated with statins to maintain mitocondrial DNA, coupled with a partial restoration of wild type tubular mitochondrial morphology.

The wild type strain transfomed with mitoGFP and treated with statins was grown in early exponential phase in glucose medium supplemented with 0,5mg/ml ergosterol (YPDE), stained with DAPI and visualized with fluorescence microscopy. As a control, in W303 rho° strain, devoid of mitDNA, only the nuclear DNA was observed. Bar: 2 µm.

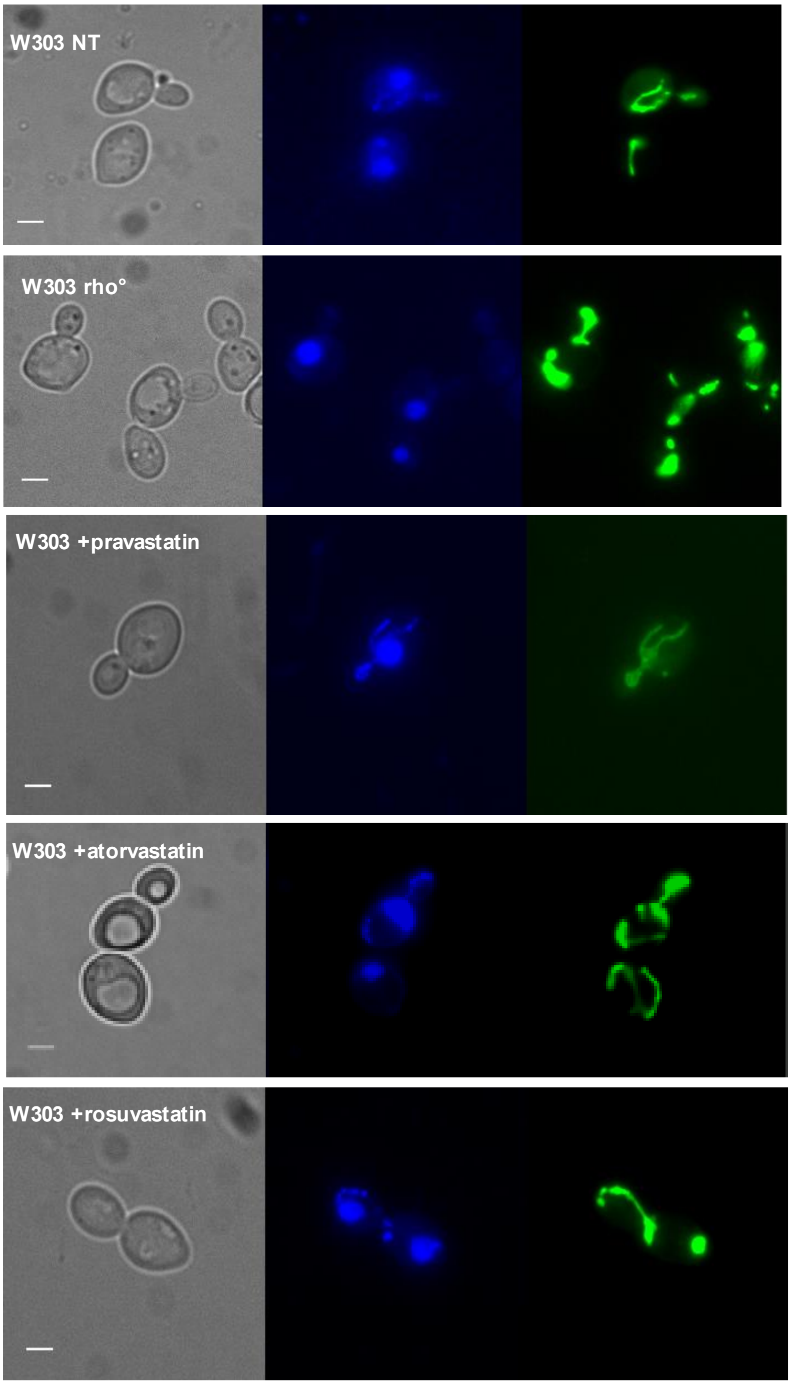

Supplemental Figure 6. Growth curves of *K. lactis* treated with statins. Statins treatments slightly affect the fitness of cells in the exponential growth phase at the reported concentrations. The *K. lactis* Δ*pda1* was used as a control, this strain lacks the piruvate dehydrogenase E1α subunit, resulting in a defect of respiration. Data are the average of 3 independent experiments.

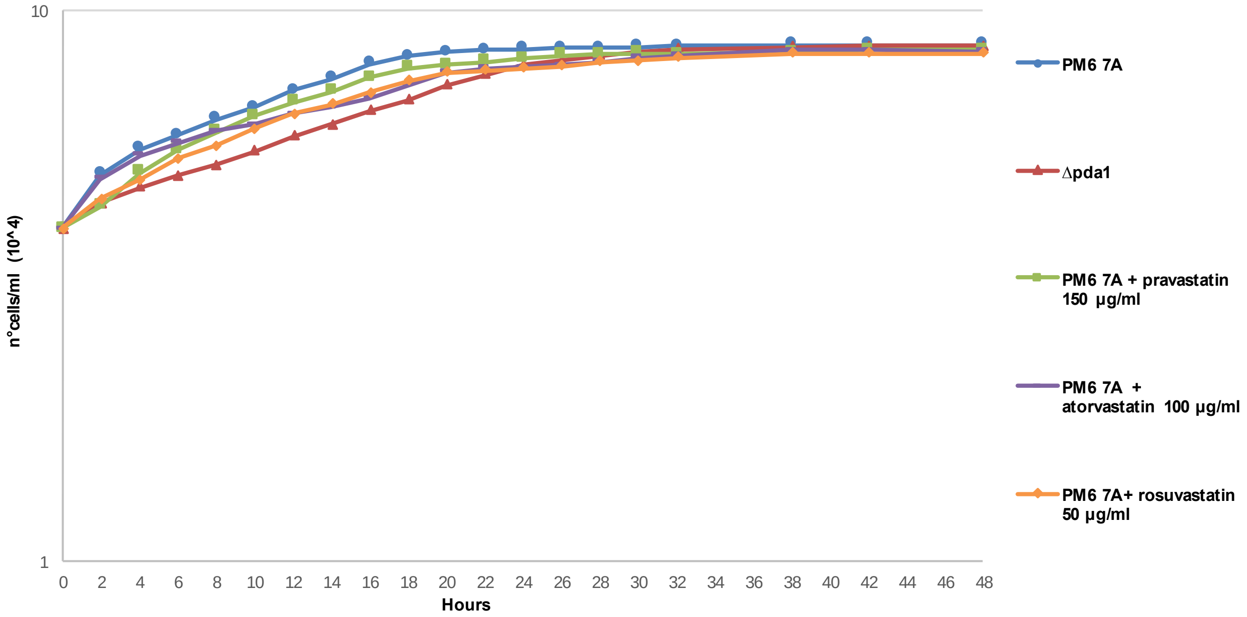

Supplemental Figure 7. Growth curves of the C25T mutant, a MELAS model, treated with statins. Statins treatments slightly affect the fitness of cells in the exponential growth phase at the concentrations used. Pravastatin induces a lag phase in early exponential phase. Data are the average of two independent experiments.

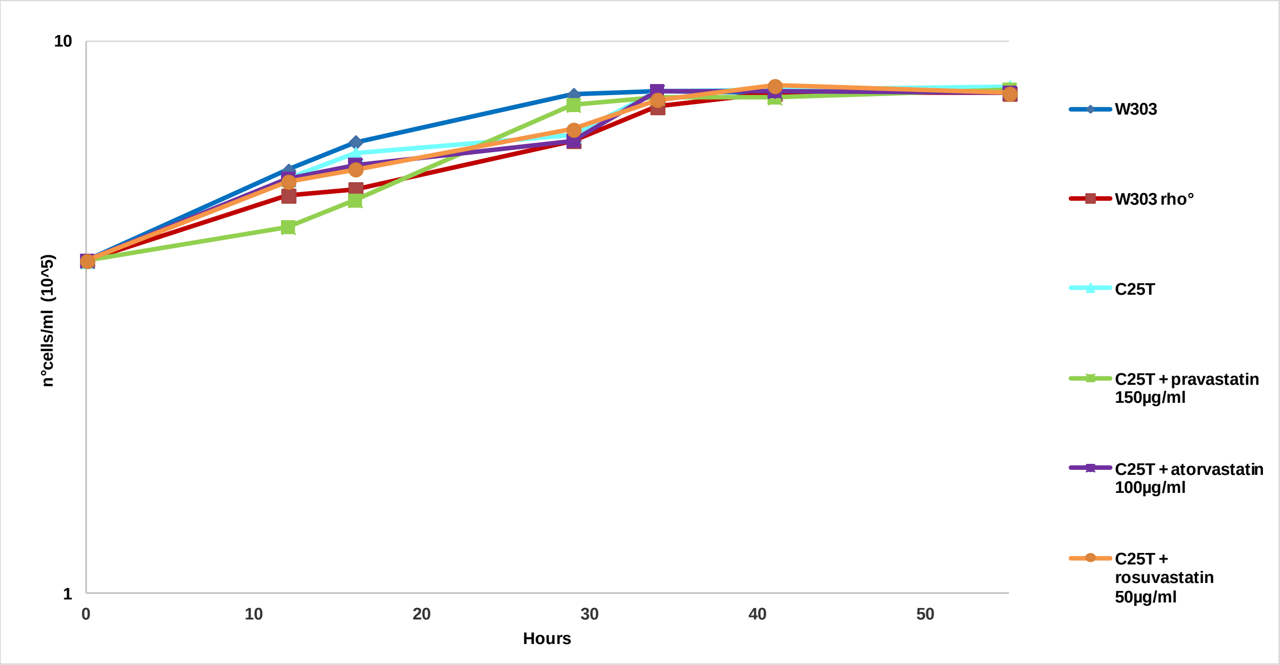

Supplement: Supplemental Material [file IENZ_A_1687461_SM6856.pdf]
